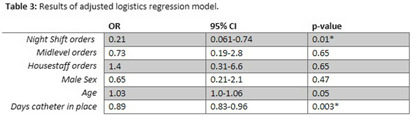# Factors Associated with Inappropriate Urine Culture Orders in Hospitalized Patients with Indwelling Urinary Catheters

**DOI:** 10.1017/ash.2024.194

**Published:** 2024-09-16

**Authors:** Ramez Azzam, Jacob Pierce

**Affiliations:** Easy Carolina University; Brody School of Medicine at East Carolina University

## Abstract

**Background:** Catheter-associated urinary tract infection (CAUTI) is among the most prevalent healthcare-associated infections. Clinical diagnosis of CAUTI and National Healthcare Safety Network (NHSN) definitions do not always align. Most patients with indwelling urinary catheters ultimately develop asymptomatic bacteriuria (ASB) due to bacterial colonization and may be misattributed as CAUTI. Urine cultures ordered on patients with ASB may lead to reporting of non-clinically significant CAUTI to NHSN. We sought to examine factors associated with ordering inappropriate urine cultures in patients with urinary catheters. **Methods:** All CAUTIs that were reported to the NHSN at a large academic medical center in Eastern North Carolina were evaluated from October 2021-July 2023. A logistic regression model was fit for patients treated for urinary tract infection (UTI) with the following covariates: age, sex, time of urine culture order, provider type, and days that the urinary catheter was in place. All data analysis was performed in SAS (SAS Institute Inc., SAS 9.4, Cary, NC: SAS Institute Inc., 2002-2023). **Results:** Table 1 demonstrates patient characteristics stratified by treatment for UTI. The analysis suggests that abnormalresults from urine cultures ordered overnight were less likely to be treated with antibiotics,and this result was statistically significant in both the adjusted and unadjusted analyses – see table 2 and 3. The model also suggests abnormal results from urine cultures ordered by housestaff and older patients were more likely to be treated for UTI, but these results were not statistically significant – see table 3. Finally, the longer a catheter was in place the less likely an abnormalurine culture was to be treated and this finding was statistically significant – see table 3. **Conclusion:** Cultures that did not prompt antimicrobial treatment did not impact patient care decisions and could be considered as inappropriate orders. This can result in CAUTIs reported to NHSN that were not clinically significant. Abnormal results from cultures that were ordered by the overnight team were less likely to be treated for clinical UTI and this may represent an important target for diagnostic stewardship interventions.

**Figure:**
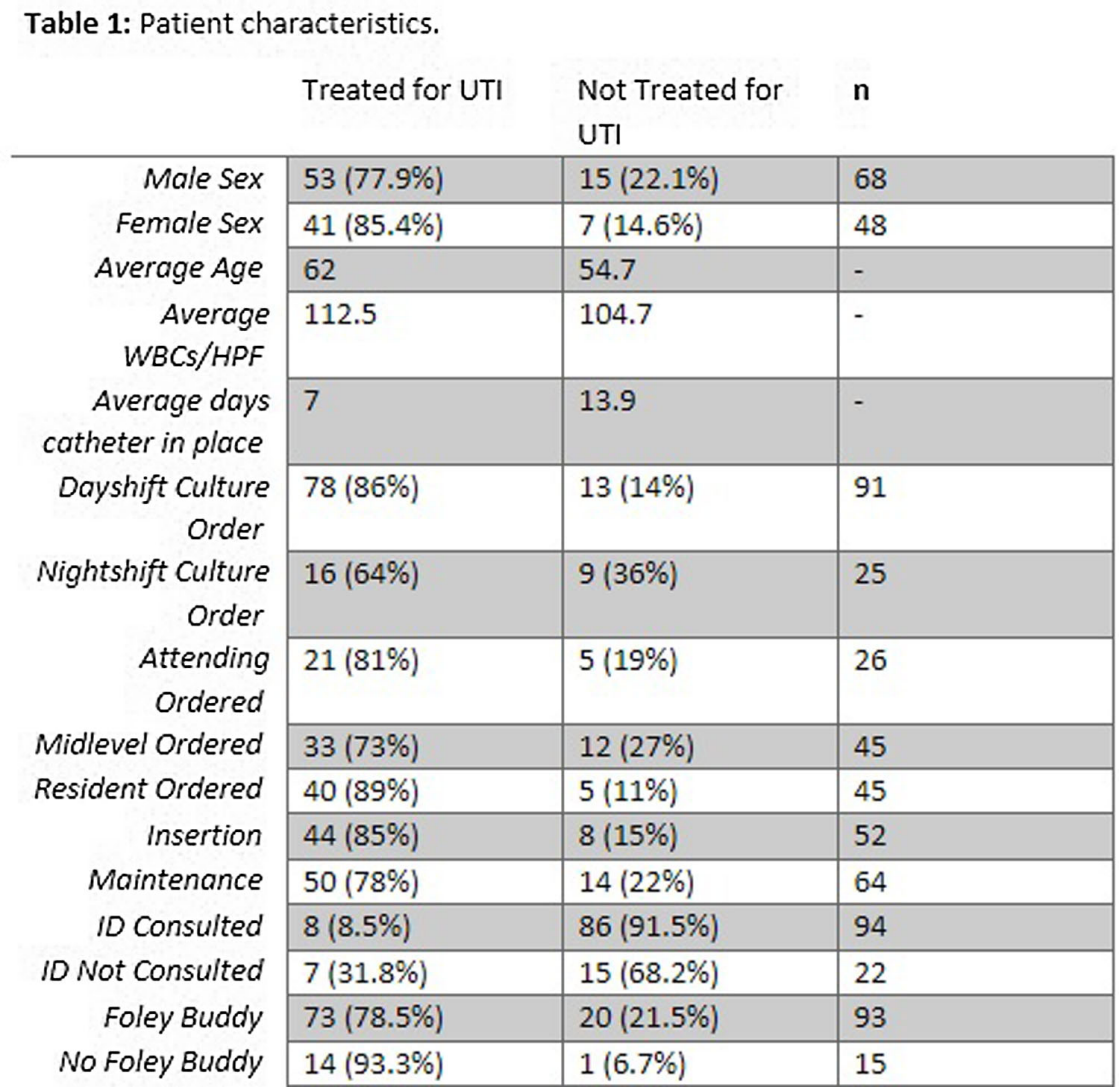

**Figure:**
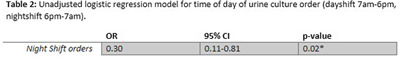

**Figure:**